# The return to work after sick leave due to common mental disorders among young employees: a qualitative interview study with young employees and managers

**DOI:** 10.1186/s12889-025-22607-5

**Published:** 2025-04-12

**Authors:** Caroline Olsson, Irene Jensen, Lotta Nybergh, Elisabeth Björk Brämberg, Helena Tinnerholm Ljungberg

**Affiliations:** 1 Institute of Environmental Medicine, Unit of Intervention and Implementation Research for Worker Health, Karolinska Institutet, Box 210, Stockholm, SE-171 77 Sweden; 2 School of Public Health and Community Medicine, Institute of Medicine, University of Gothenburg, Box 100, SE-405 30 Gothenburg, Sweden

**Keywords:** Gender perspective, Common mental disorders, Return to work, Depression, Anxiety, Stress, Sickness absence, Young male, Young female, Employers

## Abstract

**Background:**

Studies focusing on the return for young employees are scarce. The aim of this study was to investigate the return to work process after a period of sick leave due to common mental disorders among young male and female employees, with a specific focus on promoting and hindering factors. A gender perspective was applied to examine potential patterns of similarities and differences in the study participants’ experiences.

**Methods:**

A qualitative approach with an applied gender perspective was used to capture both young employees’ and managers’ experiences of the return to work process. The data consisted of interviews with 12 young male and 13 young female employees (aged 20–29) and 23 interviews with managers with experience of supervising at least one young employee during return to work. An inductive, conventional content analysis was conducted.

**Results:**

The analysis resulted in three main categories and five subcategories describing hindering and promoting factors in young employees’ return to work processes. The main and subcategories were (1) “Coordinating, planning, and timing the return to work process” (with subcategories “Lack of coordination and support from key stakeholders”, “The importance of a return to work plan and follow-up”, and “The timing of the return to work”); (2) “Gaining sufficient support and having personal resources” (with subcategories: “Level and quality of support in the work environment”, and “Resources and obstacles on a personal level”); and (3) “Poor match between the young employees’ capabilities and the work demands or working conditions”.

**Conclusion:**

The study participants identified similar factors that promoted and hindered the return to work process. Young employees need more support from employers and various welfare agencies. Managers need to know more about the return to work process and the responsibility of involved stakeholders. No expressed gender differences were identified.

## Background

Sick leave due to common mental disorders (CMDs), including depression, anxiety, adjustment disorders, and stress-related ill health [1], is a major problem for individuals, organisations, and society at large [2]. In Sweden, CMDs are a more common cause of sick leave experienced by women than men. According to the Swedish Social Insurance Agency (SSIA), mental disorders account for 50% of all women’s sick leave, while the corresponding number for men is 39%. Among young employees aged 16–29, CMDs are the most common cause of sick leave [3]. Since young employees have a long working life ahead of them [4], the high sick leave rates due to CMDs among young adults [3] are a matter of concern. Young age has been shown to be a predictor of successful return to work (RTW) [5–7], although there is some evidence to the contrary [8]. In addition, little is known about young employees’ experience of RTW.

In Sweden, employees on sick leave for more than seven consecutive days are required to provide a medical certificate to their employer. The employer has an obligation to pay sick pay during days 2–14 (about 80% of the regular salary), and thereafter, the employee can apply for sickness benefits from the SSIA. Swedish employers play an essential part in RTW and have several obligations; for example, if the sick leave period is expected to be longer than 60 days, the employer needs to make an RTW plan and list possible work adjustments that could promote RTW for the sick-listed employee. When the SSIA assess if an employee can receive sickness benefits, it compares the employee’s work capacity to his or her regular work (during days 15–90); thereafter, the work capacity is compared to adjusted work at the same employer (days 91–180). After day 180, a sick-listed employee can only receive sickness benefits if the SSIA assesses that he or she cannot perform any work that exists in the general labour market, but there are exceptions to this rule [9].

Research has suggested that RTW should be seen as a process rather than a one-time event [10–12]. To understand how young employees can best return after sick leave [13], it is relevant to examine their experiences before, during, and after RTW. As a concept, RTW is not always clearly defined; in most studies, RTW implies a return to the same employment [13]. However, given that young employees often have insecure or temporary forms of employment, this is not always possible [14, 15]. Young adults may not want to return to their current position or employer. Many of them are still deciding on a career and, after a period of sick leave, may decide to change paths altogether.

The Swedish labour market is gender divided, where male and female workers work in different sectors [16, 17], which also implies that they are exposed to different health risks at work [18]. Gender norms are also produced and upheld in workplaces [19, 20]. Research on the RTW process seldom takes aspects of gender into account. Of the few studies that have included a gender analysis, two highlight the significant pressure placed on female workers both at work and in their private lives [21, 22]. Parenthood and other social responsibilities that women carry more than men do add to the burden of work-related factors [23]. Furthermore, since previous studies have shown that an individual’s private life can influence their RTW process, this study sought to collect data about both work and private life [12, 21].

This study is part of a larger project aimed at investigating the experience of sick leave due to CMDs and the process of RTW for young employees from the perspective of young employees and managers (see the published study protocol [24]). Some findings from the collected data have already been published, such as an examination of the perceived causes of sick leave, as described by young employees and managers [25, 26]. In the present study, the focus is on the RTW process.

The aim of the current study was to investigate the intended return to work process after sick leave due to common mental disorders among young male and female employees, with a specific focus on promoting and hindering factors, as experienced by young employees and managers. A gender perspective was applied in the analysis to examine patterns of similarities and differences regarding the young male and female employees’ RTW processes.

## Methods

### Design

This study adopted a qualitative approach that allowed participants to describe their experiences and thoughts on RTW after sick leave due to CMDs in a semi-structured interview [27]. A gender perspective was used to ask questions about and examine potential similarities and differences between young male and female employees’ RTW processes.

### Sample and setting

The study participants were recruited from across Sweden. We used a purpose sampling strategy aiming to include study participants with experiences that could generate rich and nuanced descriptions regarding young employees’ RTW after sick leave due to CMDs [28]. To be included, the sick leave period in focus was set to 3–12 weeks. This criterion was chosen to align with the period in which the employee’s work capacity is assessed by the SSIA and compared with his or her regular work or adjusted work at the same employer. This, and the other inclusion and exclusion criteria, are shown in Table 1.

**Table 1:** Inclusion and exclusion criteria

Employees	Managers
Inclusion criteria: aged 19–29; currently, or within the past two years, on sick leave for 3–12 weeks due to CMDs.	Inclusion criteria: First-line managers with ≥ 1 year managerial experience and working ≥ 50%; line management responsibility during the past years for young employees (19–29 years) on sick leave due to CMDs for 3–12 weeks.
Exclusion criteria: posttraumatic stress syndrome, neuropsychiatric diagnoses, psychotic symptoms, and bullying problems; persons without employment or employed by staffing agencies.	Exclusion criteria: Managers investigated for workplace misconduct, including harassment or abuse.

Two interview guides were constructed to capture the experiences of RTW for young employees with CMDs, and these guides have been published as supplementary material for our study protocol [24]. The interview guides were designed to collect data on clusters of questions in the following order; causes of sick leave, promoting and hindering factors in the RTW process, and the potential impact of gender, young age, and the Corona pandemic. The interview guides were tested in four pilot interviews, two with employees (one young male employee and one young female employee with experience with sick leave due to CMDs) and two with managers (one male and one female). The purpose of these pilot interviews was to test the questions and their comprehensibility for the respondents, so data from these interviews were not included in the analysis. Only minor changes were made to the interview guides after these tests. All interviews were conducted via Teams, Zoom, or telephone between November 2020 and August 2021 by the first (CO) or last (HTL) author, both of whom hold a PhD and are trained and experienced in conducting interviews from several previous qualitative research projects. The interviews were transcribed verbatim by a professional company and then pseudonymized and cross-checked for accuracy.

### Participants

When designing the study, the authors estimated that approximately 20–25 participants in each group would be manageable and sufficient to gain data rich enough to fulfil the aim of the study. During data collection, the authors had continuous discussions and evaluations of the collected data and considered data sufficient after having interviewed 25 young employees (12 males and 13 females) and 23 managers (7 males and 16 females). All participants were asked to state their self-perceived gender identity. None of the study participants identified themselves as nonbinary or as having a gender other than male or female. There was no connection between the young employees and the managers. The participants represent a range of different work occupations and sectors. The age range for the young employees was 20–29 years, and for the managers, it was 36–61. The participants’ characteristics are shown in Table 2.

**Table 2:** Participant characteristics

	Young employees		Managers	
	Female	Male	Female	Male
Number	13	12	16	7
Age in years, mean, (range)	27 (20–29)	27 (22–29)	48 (36–61)	50 (44–59)
Occupational sector*^#^				
Health care/Social service/Education	6	3	11	1
White collar	5	3	5	2
Service	2	1	2	1
Blue collar	0	5	0	3
Years of experience as managers				
≤ 5 years			3	1
6–10 years			6	1
≥ 11 years			7	5
Years at current workplace				
< 1 year	2	6		
1–2 years	5	2		
3–5 years	6	3		
6–10 years		1		
Levels of education				
Primary/Secondary education	4	8		
Higher education/University	9	2		

*Two female managers had managerial experience from more than one occupational sector and are represented in multiple rows. ^#^The Occupational sector refers to a broad categorisation. Examples of occupations in the heading white collar are in our study management consultant, accountant and IT-developer. Examples of blue-collar occupations are factory worker, building caretaker and construction worker

### Data analysis

The collected data were analysed using inductive conventional content analysis [29]. NVivo software was used to structure the coding. The interviews with the young employees and managers were analysed separately. The two analyses of the transcripts with the young employees and managers followed the same steps. First, the analysis began with all authors reading several interview transcripts and having an initial discussion. CO and HTL read all the transcripts several times. To reach a shared understanding of the material and the coding structure, the first interviews (five with young employees and four with managers) were coded collectively by CO and HTL. The remaining interviews were coded independently by CO or HTL, but with continuous discussions. Short descriptions of each interview were written directly after coding to summarise them and contribute to an understanding of how the codes were linked. During this process, the research team met several times to discuss the results and improve credibility [30].

For this study, data regarding the RTW process was selected and analysed, the parts of the interviews relating to causes of sick leave and the impact of the Covid pandemic had previously been analysed and published elsewhere [25, 26]. During the analysis process, the codes relating to RTW were merged into subcategories, and the subcategories were then further abstracted into main categories until the categories became nonoverlapping. In parallel, reflective annotations were made regarding gendered patterns in the main and subcategories.

## Results

The analysis revealed three main categories and five subcategories (see Table 3). Most of these categories include factors deemed to both promote and hinder the return to work process. Each factor is marked with an E for employee, an M for manager, or both if both groups identified it as hindering or promoting RTW.

**Table 3:** Main categories, subcategories, promoting factors and hindering factors

Main category	Subcategory	Promoting factors	Hindering factors
Coordinating, planning, and timing the RTW process	Lack of coordination and support from key stakeholders	Involved stakeholders fulfilling their obligations (E & M)	Lack of knowledge of different stakeholders’ responsibilities and roles (E & M) Troubles when navigating the bureaucracy surrounding RTW (E & M)
	The importance of an RTW plan and follow-up	Following the jointly decided rehabilitation plan (E & M) Taking a parental approach (M)	Lack of RTW plan Lack of adherence to the RTW plan by the manager (E) or the employee (M)
	The timing of the return to work	Involving the young employee when deciding when to RTW (E & M) Gradual return (E & M)	Difficulties in knowing when to RTW (E & M) Too slow or too fast RTW (E & M)
Gaining sufficient support and having personal resources	Level and quality of support in the work environment	Making work adjustments (E & M) Having understanding colleagues (E & M) Keep in contact with the workplace (E & M) Good communication and dialogue between employee and employer (E & M) Having access to OHS to help plan adjustments and navigate RTW bureaucracy (M)	Troubling when extra work is assigned to colleagues of the young employee (E & M) Worries related to perceived expectations that the young employee has the same work capacity as before (E) Lack of possibilities to initiate work adjustments (E & M), especially for small organisations (M)
	Resources and obstacles on a personal level	Good social relations outside of work (E & M) Adding recovering activities (E) Access to medication (E & M) Time to recovery during SA/gradual RTW (E) Setting realistic goals (E & M)	Poor social relations outside of work (E & M) Troubles realising own limits and handling high ambition during RTW (E & M) Stigma and shame about CMDs (E & M) Financial worries (E)
Poor match between the young employees’ capabilities and the work demands or working conditions		Change of career path or work position (E & M)	Poor working conditions (E) Feeling that the manager failed the employee during the RTW (E) Too stressful work demands (E) The young employees’ capacity is not matched with work demands (M)

### Coordinating, planning, and timing the RTW process

This main category captures young employees’ and managers’ experiences with the welfare systems and regulations involved in rehabilitation. It is divided into three subcategories: Lack of coordination and support from key stakeholders; The importance of an RTW plan and follow-up; and, The timing of the return to work.

#### Lack of coordination and support from key stakeholders

Both young employees and managers described a lack of previous experience of being in a rehabilitation process, which meant that they lacked knowledge of the different stakeholders’ responsibilities. Still, they needed to navigate in this, for them, new landscape. Both managers and employees stated that the key stakeholders, such as the Social Insurance Agency and healthcare system, could promote RTW when they were fulfilling their roles and hinder it when they failed in their obligations. The bureaucracy and the many contacts with the key stakeholders were more often considered as hindering rather than facilitating RTW.

When there was a medical doctor who took care of the individual and helped the person in a good way so that they got follow-up meetings, the right medicine that worked and so on, then you had a dialogue. And we had good exchanges, we had meetings every other month to check that everything was working well. And then it worked quite well for over a year and a half without any major catastrophe, you might say. But when that doctor…//…disappeared, then someone else was supposed to take over and then it fell through the cracks and there was no follow-up. (IP 11, male manager, white collar).

The young employees described situations where despite suffering from their CMD symptoms, they still had to navigate welfare systems that they found overwhelming. They described how they were unable to get in contact with their medical health care centre or could not understand what the Social Insurance Agency expected from them.

My boss messed up with my benefit application, so I didn’t get any money there either. And I tried… Because I had… The psychiatric doctor I’d been assigned to at my local health centre, he was always fully booked, so you could only get to see him every two or three weeks. And then he had to take a break, because his job was so intense… When we were supposed to meet, he often didn’t show up because he had to stay home and look after his kids when they were sick. At the time I knew that I could try to see a counsellor at the health centre, but they were also fully booked. (IP 18 young male, chef, service)

Managers also highlighted as problematic the bureaucracy surrounding sick leave and RTW with many procedures to keep track of for persons ill with a CMD. To promote RTW, some managers went beyond their formal responsibilities, helping their young employees contact their doctor, the Social Insurance Agency, or other authorities.

#### The importance of an RTW plan and follow up

On an organisational level, both young employees and managers felt that it was important for promoting RTW to set up an RTW plan and then follow it. The study participants thought these plans worked well most of the time. In some cases, there was no RTW plan, or it was not followed. When young employees described negative experiences with a RTW plan, they often pointed out a lack of commitment from their manager as the problem, whereas for managers, negative experiences occurred when young employees did not follow the agreed-upon plan.

So I worked really hard to come back, but she [the manager] didn’t even have any meetings with me about this, that is, meetings about how I might come back to work. That was really strange. So… yes, I don’t really understand how she managed my return, actually. (IP 7, young female, occupational therapist, health care)

For managers, the challenge was that, due to medical confidentiality, they did not always have all the facts regarding their employees’ medical situations. Managers also expressed uncertainty about their responsibility towards employees on sick leave and the levels of support they, as managers, could expect from, for example, their employers’ occupational health services.

Who is actually responsible for ensuring that the person will be fit for work again? That responsibility is not clearly laid out. It is clearly stated that the company should do as much as they can to help and that the person should do as much as they can, but who is responsible for helping the person? Because the person in the situation can’t be fully responsible for it themselves. In any case, it’s something like that, I think. (IP 13, female manager, white collar).

Managers described how young employees, due to their young age, had limited life experience, which added to the difficulties for them in understanding and handling the RTW process. As a consequence, managers felt that they needed to provide extra guidance and care for young employees. Some managers even described this as taking a parental approach, which meant more regular phone calls or meetings about not only work but also personal life matters that affected the employee during the RTW.

#### The timing of the return to work

Both young employees and managers felt that time was an essential element of RTW. Finding the right time for employees’ first RTW and how gradual their return to their previous employment level were both factors perceived to promote RTW. Participants agreed that it was essential that the young employee was involved in decisions regarding the extent and pace of their return. However, employees and managers felt it was difficult to know how fast or slow the RTW should be. They feared, on the one hand, that an RTW that is too early could be a risk factor for relapse. On the other hand, some young employees voiced their frustration when they perceived that their RTW process was progressing too slowly. This perceived delay led to increased anxiety and worries about RTW. A gradual reintroduction to the workplace set at the right pace could often mean working initially for two hours a day (25%), then 50%, and 75%, before finally working full time again. Both young employees and managers saw this gradual increase in work time as a factor that promoted successful RTW. While managers and employees both perceived gradual RTW as beneficial, young employees often thought that RTW was too slow. In contrast, managers argued that the RTW process needed time to reduce the risk of relapse.

Because my experience is that it usually goes too fast. And then I’m the one who has to put on the brakes. And then I say, “No, let’s leave it there for now. 75% is just fine. I don’t think you should come back to work anymore than that, let’s just stick with this arrangement for a while.” (IP 6, male manager, education).

How long the period of full-time sick leave should be and when in time the RTW process should start, was also brought up by study participants. Both young employees and managers experienced that it was difficult to know when it was optimal. Even if the return date was hard to decide upon, it was still described as essential to consider and to discuss with all stakeholders (employees, managers, occupational health services, health care providers, and the Social Insurance Agency) to promote successful RTW.

### Gaining sufficient support and having personal resources

This main category captures the experiences of different forms of support that the young employees in this study expressed as promoting or hindering their RTW. The two subcategories are level and quality of support in the work environment and resources and obstacles on a personal level.

#### Level and quality of support in the work environment

Support from a manager included work adjustments and social arrangements. Both young employees and managers felt that maintaining a degree of social contact with the workplace during sick leave helped to promote RTW. For managers, these social contacts were seen to promote successful RTW. Social contact was described as an important way to contribute to their employees’ well-being and show them they were valued. Managers stressed that they needed to be available and listen to their employees. When employees return to the workplace, managers often try to stay in touch with them more often to check their health status and help them organise their work tasks.

Booking a time to meet, talking to them, chatting a bit, meeting occasionally, I think that’s crucial too, so that they don’t get left behind. It’s really important that they have this contact, that there is this connection, even if they are on full-time sick leave, that you keep in touch so that you don’t lose them, rather that they feel they’re important and that you want them to come back. At the same time there needs to be a balance, because this shouldn’t be too demanding, so that they feel stressed or under pressure. (IP 7, female manager, social services)

Young employees mentioned the importance of being encouraged by their manager. The manager could arrange fewer work tasks for the returning employees, facilitate their reduced working hours, and provide a supportive work environment where they could talk about their CMDs with understanding colleagues.

I feel that I can be more open, that if I feel that this isn’t working or I’m starting to feel bad, I can say so. It may be that I know my colleagues better, but also that during the time I was ill, I felt that… it’s… A lot of what caused my anxiety to increase was that I kept it inside me. And if there was a problem, I didn’t talk about it, but it was also inside me. So that’s when I thought that if I talked about it, it would be… even if it was just something small. If I bring it up with a colleague, it might be easier. And it has worked. (IP 8, young female, debt collector agent, white-collar worker).

Managers’ and colleagues’ open attitudes toward CMDs were considered to contribute to well-being and enhance the likelihood of RTW. Nevertheless, it was troubling for some managers and young employees when they noticed that extra work tasks were assigned to the young employee’s colleagues because of their absence or reduced work capacity.

Furthermore, perceived expectations from colleagues after full RTW could lead to mental pressure for young employees. The young employee worried that colleagues would assume that they could work to the same extent as before their sick leave. The managers described that it was sometimes a challenge to know how much to reveal to the work team about their colleague who was currently in an RTW process. Furthermore, managers reflected upon how much understanding they should expect from the young employees’ colleagues when they had to take on extra work to reduce work pressure for the person in RTW.

Managers and young employees agreed that work adjustments were often necessary to return to work. Limiting workload, reducing hours, and recovery time during the workday were measures described that helped to promote RTW. However, young employees in this study also reported incidents where the manager did not always manage to adjust work to the necessary level.

A person who […] is coming back to work should be given jobs that aren’t too pressured with short deadlines. But I’ve had deadline after deadline after deadline after deadline and practically zero control over what I’m doing, and I’m trying to learn at the same time and yes, my colleagues who work with me don’t always have time [to help me out]. They’re caught up in other projects and have just been thrown into this, too, so it just has to be done. So that’s what I’ve been struggling with and am struggling with right now. (IP 21, young male, business analyst, white collar-worker).

Economic priorities and lack of time were common limitations described by the managers as hindering RTW. Handling young employees with CMDs was not always easy for managers, and they stressed the importance of receiving their own support in the RTW process. Even if managers in this study reported that they frequently felt supported by their human resources department (HR) and/or external experts, they meant that only large organisations had a range of tools available for support. This included using external occupational health services or private therapeutics, support from an HR department and the opportunity to redeploy an employee to another department or find alternative work tasks for them within the organisation.

I think that companies need to prepare themselves for this type of issue. With specialists who can support the managers. Because we have to do a lot - we have to, well, it’s really me who handles the whole case, and it takes a lot of time. And if you’ve never done it, it’s a ton of work. So that the companies actually organise all of this centrally, and the occupational health service understands what the manager needs. Of course, the person on sick leave also has needs, but the manager also needs to understand and get support. Because we have, I mean, I have the ultimate responsibility. (IP 5, male manager, blue collar).

According to managers, access to occupational health services meant limiting their involvement in their young employee’s symptoms and focusing on ensuring they had the support necessary to promote their RTW. Managers also said they needed more information about the rules and regulations on workers’ rights and the social welfare system.

#### Resources and obstacles on a personal level

A supportive personal life was a central part of the recovery process for a young employee returning to work, according to both groups of participants. Good relationships with a partner, friends, or family were described by both managers and employees as important informal support networks that promoted the RTW process.

I’m really grateful that I’ve got such good friends as I do. They’ve helped me so much. I think I’ve been pretty lucky in that I’ve got a lot of sensible friends who understand, who you can talk to. (IP 22, young male, building caretaker, blue-collar worker).

Other forms of personal support that could promote RTW included medication, mindfulness, yoga, and exercise. A few young employees in this study had an ambiguous attitude toward medication, meaning that they felt that medicine might work but could have other negative effects. Managers sometimes witnessed how young employees did not want to take prescribed medication for their CMDs, which, according to the manager, could hinder their RTW process. On the other hand, some of the young employees said that medication had contributed to their recovery. Young employees also described the sick leave period per se as essential for giving them time to recover.

Young employees and managers both said that work-related CMDs and the sick leave that can result came about because of the high ambitions many young people had. Both managers and young employees expressed that young employees need to realise and understand their limitations if RTW is to be successful. They need to initiate personal changes and reduce the expectations they place on themselves. Both managers and young employees saw the ability to recognise and change behaviour in relation to demands and levels of ambition as an important personal resource in a successful RTW process.

To have more realistic goals. Or not putting too many demands on yourself. Because at the end of the day, I seem to be the only one who’s putting these demands on myself [laughs]. So I’m trying to get better at being more realistic and being fair to myself. And when I can’t do things, to say that I can’t and try to learn how. Or, to simply just let it be. And it has worked, it has gotten better, yes. (IP 12, young female, afterschool leisure teacher/assistant nurse, education/health care).

The young employees also described feelings of stigma and shame arising from their CMDs. This phenomenon hindered the RTW process. In addition, managers, wishing to maintain young employees’ integrity, sometimes found it difficult to know how much they could interfere with employees’ personal problems. When, in some cases, a young employee had personal issues, managers offered help, even when the problems were not work-related.

Young employees found it stressful to be on full or partial sick leave because of their reduced income. They expressed the need to balance the pace of their RTW with a perceived risk of short- or long-term economic consequences. Some young employees expressed a fear of never being able to work again and worried about their future:

You disappear into your thoughts and just start with the “but will I ever work again? Will I ever be normal? What happens if I get kicked off social security? What…” I have no idea… I still don’t know what… If I was to be sick for more than a year, would I lose my job and be expected to look for low-level job that doesn’t exist where I’ll just stand and stack shelves? So you feel pretty vulnerable. (IP 21, young male, business analyst, white collar-worker).

Personal resources were essential for successful RTW. The study participants felt that financial worries and personal demands, such as caring responsibilities or poor personal relationships, hindered the RTW process.

### Poor match between the young employees’ capabilities and the work demands or working conditions

Even though the intended outcome in an RTW process is supposed to be return to the same employer, some managers and young employees described a poor match between the young employee and the work demands or working conditions. However, the managers and the young employees had different understandings of the reason. While the young employees highlighted the poor working conditions, managers, on the other hand, emphasized the poor match of the young employees’ capabilities with the required work demands for the specific job position.

Some of the young employees thought that their employer had failed them during the period preceding their sick leave and during the RTW process. As a consequence, they felt that they needed to leave the specific employment and, in some cases, even the work sector or profession. As an example, some young employees described how their manager had failed to set reasonable limits on their workload before they went on sick leave and failed to provide sufficient support or adjustment when it was time to RTW. Some said that they needed to leave the profession or work sector because of their experience with a stressful work environment that they associated with the work role in question. Deciding to change career paths was described as generating feelings of loss. These young employees were forced to give up a job they had initially enjoyed and cherished.

No, ugh. I’m never going back to restaurant work again. It’s not on my personal roadmap. Instead, I’m focusing on this [a new job in a different work sector] and a different kind of future. (IP 18 young male, chef, service).

Some managers described situations where they had tried to help young employees on sick leave to RTW, but sometimes their efforts had not been enough. For these managers, this failure was understood as the result of poor job matching, meaning that the young employee was never the right person for the job or that the work tasks were too difficult for them.

Then maybe this person didn’t fit here either. Maybe they have the wrong profile. So it goes all the way from recruitment to introduction, to being able to perform at work. [---] I think when you’re a manager, there’s a difference between someone who’s worked for a long time and gets ill, and someone who’s fairly new to the job and doesn’t really manage it. Yes, and if it’s a probationary period, then you just end the employment. (IP 2, female manager, service and white-collar).

Some managers also thought that some young employees would benefit from not coming back to the work position they had when they went on sick leave.

The return process, I’ve experienced it so differently with each individual, but I can say that it’s usually quite difficult, in my experience, to get back to the position and status you had when you initially burned out. It can be hard to get back into the same organisation again, where you think… often there’s a feeling that there’s a lot of chitter chatter going on behind your back, you see yourself as a big failure. (IP 10, female manager, white collar).

In some cases, managers expressed that it was unfortunate that it had to take an incident of sick leave for the young employee to realise that the work position was not a good match for him or her. In these cases, managers thought it could lead to an opportunity to make new career choices early in life.

## Discussion

We found several common hindering and promoting factors expressed by managers and young male and female employees regarding the RTW process; some of these factors were considered more severe due to the employees’ young age. Interviewed managers and young male and female employees shared the experience that the rehabilitation process was insufficient and difficult to navigate. When returning to work, young employees expressed that they lacked knowledge of what to expect from managers and were left without guidance from healthcare bodies and the SSIA. Moreover, young employees’ worry about becoming unemployed and falling out of the social welfare system hindered their recovery and RTW.

The impact of age was evidenced by some managers describing how they took more responsibility and went beyond their job description to deal with young employees compared to their management of the RTW process for more experienced and older employees. They described their efforts as a form of parenting, indicating that young employees needed more guidance on how to manage the RTW process, including the functions of the involved stakeholders (employers and managers, health care providers, and the Social Insurance Agency). According to Swedish law, it is mandatory for regions to provide rehabilitation coordination for sick-listed patients to facilitate RTW. However, our study participants described a lack of coordination between the employee, the manager, the health care system, and the SSIA and experienced this as a hindrance to successful RTW. As research has shown, employees on sick leave need predictability and a sense of control over the situation [11]. For young employees with little work and life experience, these features may be even more crucial. The study participants considered it an extra burden when it was difficult to contact those responsible for treatment from the health care provider. A lack of adequate treatment has been shown to have possible serious long-term implications; for example, one longitudinal study has shown that young adults (19–29 years) with CMDs have a greater risk of experiencing a new CMD episode ten years later [31].

A few differences in the perspectives of the managers and young employees regarding the hindering and promoting factors were also found. According to the Swedish model, employers are responsible for making an RTW plan and suggesting work adjustments during the RTW process; however, our study showed that it was sometimes difficult for managers to live up to their responsibilities. This could lead to the young employees feeling that the manager did not provide sufficient support to promote their RTW. It was clear that managers felt limited by their organisational context. Being obliged to limit support because of time or financial constraints and prioritising productivity before employee wellbeing is an aspect of sick leave management that has been described previously [32, 33]. Since successful RTW can take a long time, as demonstrated by Nielsen and Yarker [34], adjustments may be needed for years to come. In line with previous studies [35, 36] the interviewed managers with experience from work in small organisations, found it even more challenging due to limited resources compared to lager organisations. They describe how they had fewer options to redeploy employees, adjust work tasks, and sometimes lacked organisational support in their role as managers.

Previous studies spanning over two decades have reported gender differences regarding promoting and hindering factors for RTW [12, 21, 23, 37], it was therefore unexpected that our study participants only expressed gender differences concerning causes of sick leave [25, 26] and not regarding the RTW process even if the order of the interview questions made it possible. Previously identified gender differences have mainly been related to the higher levels of domestic strain experienced by women and the intensification of strain for women with children [22]. Parenthood was not mentioned by our study participants as impacting the RTW process for young employees. That no gender differences were expressed by the study participants could be understood as meaning, with the best of intentions, that RTW is a process designed to encompass employees’ needs regardless of their gender. Or it could be understood, in a less favourable interpretation, as a sign of gender blindness. According to the WHO’s Gender Responsive Assessment Scale [38], gender-blind policies and practices ignore gender norms and roles and are considered less favourable outcomes than gender-sensitive, equal or transformative policies and practices. If it is a matter of gender blindness in this case, such blindness could imply a risk that young male and female employees’ various needs and conditions are overlooked.

## Implications

Although the Swedish laws and regulations regarding RTW state what responsibilities involved stakeholders have, our findings indicate that the RTW process is not clearly understandable and transparent for young employees on sick leave, nor for managers. In Sweden, it is mandatory for organisations to have policies and guidelines on how to proceed in the RTW process. Our results implicate that managers have difficulties implementing these in the RTW process for young employees. Information about the social security system and about financial compensation when on sick leave should be easily accessible. For young adults, access to affordable healthcare is also essential, as illustrated by our findings in the current study and in a previous study conducted in a Norwegian context [31]. Moreover, knowledge about requalification initiatives might be beneficial for some young employees for whom a return to the same workplace or occupation is not considered a sustainable option.

Our results also have implications at the organisational level. Some of the managers we interviewed had only basic knowledge of the RTW process and what their responsibilities were supposed to be. Other managers with more advanced knowledge reported on the organisational and financial restrictions they faced when trying to support a young employee’s return. These results suggest that managers need more knowledge and more organisational resources to facilitate RTW, especially when employees are young. Organisational resources are crucial to sustainably relocate work tasks and to ensure a work environment where sick leave is not shaming or where CMDs are stigmatised. Given the high prevalence of CMDs among young employees and the fact that they should have a long working life ahead of them, future studies are needed to investigate whether a well-functioning rehabilitation plan for young employees could facilitate RTW and, if so, how it might benefit from increased gender awareness.

### Strengths and limitations

To improve the quality and credibility of the studies, the Coreq checklist for qualitative research was used [39]. One strength of this study is that young employees with an experience of sick leave due to CMDs were interviewed alongside managers who had supervised at least one such person. That there was no direct link between the managers and the employees can be seen as both an advantage and a disadvantage. The benefit is that the young employees could talk openly about their work difficulties and other subjects they wished to keep confidential from their manager. The disadvantage is that the experiences are not linked and refer to different events.

The interview guide was designed to capture aspects that the interviewee thought were related to young age or gender, and several gender differences were reported in the interviews regarding the causes of sick leave [25, 26] but not concerning the RTW process. The methodological action to take when no gender differences are empirically found, even though a gender perspective has been applied in the analysis, is, as Tannenbaum et al. points out, to report such results [40]. The order of the interview questions aimed to capture the interviewees’ perspectives on gendered aspects in the whole process, from causes to sick leave to intended return to work. It should be recognised that the results might have differed if the interviews had only covered the RTW process.

## Conclusion

Our results indicate that young male and female employees need more guidance in navigating the RTW process than older, more experienced employees. They often lack sufficient knowledge of the support they can receive from their managers, healthcare, and welfare agencies. Furthermore, managers highlighted a need to take a parental approach when supporting young employees’ RTW processes. A difference in perspective was found regarding the reasons behind an identified mismatch between young employees’ capabilities and work conditions and demands. Managers also need more knowledge and organisational support to be able to provide guidance and fulfil their responsibilities. No gender differences were expressed by either the managers or young employees.

## Data Availability

The datasets generated and analysed during the current study are not publicly available due to Swedish ethical review regulations. The data are available upon reasonable request. Inquiries for data access should be sent to Karolinska Institutet, Institute of Environmental Medicine, Unit of Intervention and Implementation Research for Worker Health, Box 210, 171 77 Stockholm or to the principal investigator Caroline Olsson, caroline.olsson@ki.se, who will then contact the Swedish Ethical Review Authority for permission to openly share the data.
